# Diaqua­bis­(5-carb­oxy-2-propyl-1*H*-imidazole-4-carboxyl­ato-κ^2^ *N* ^3^,*O* ^4^)nickel(II) tetra­hydrate

**DOI:** 10.1107/S1600536810025237

**Published:** 2010-07-07

**Authors:** Run-Zhen Fan, Shi-Jie Li, Wen-Dong Song, Dong-Liang Miao, Shi-Wei Hu

**Affiliations:** a College of Science, Guang Dong Ocean University, Zhanjiang 524088, People’s Republic of China; b College of Food Science and Technology, Guang Dong Ocean University, Zhanjiang 524088, People’s Republic of China

## Abstract

In the title complex, [Ni(C_8_H_9_N_2_O_4_)_2_(H_2_O)_2_]·4H_2_O, the Ni^II^ ion is coordinated in a slightly distorted octa­hedral environment formed by two bis-chelating H_2_pimda (H_3_pimda is 2-propyl-1*H*-4,5-dicarb­oxy­lic acid) ligands and two coordinated water mol­ecules. In the crystal structure, a three-dimensional framework is formed by inter­molecular O—H⋯O and N—H⋯O hydrogen bonds involving the solvent water mol­ecules, coordinated water mol­ecules, carboxyl­ate O atoms and the protonated N atoms of the H_2_pimda ligands. The propyl groups of each H_2_pimda ligand are disordered over two sets of sites with refined occupancies of 0.50 (2):0.50 (2) and 0.762 (11):0.238 (11). In one water solvent mol­ecule, one of the H atoms was refined as disordered over two sites of equal occupancy.

## Related literature

For the potential uses and diverse structural types of imidazole-4,5-dicarb­oxy­lic acid complexes, see: Zou *et al.* (2006 ▶); Li *et al.* (2006 ▶); Liu *et al.* (2004 ▶); Sun *et al.* (2005 ▶).
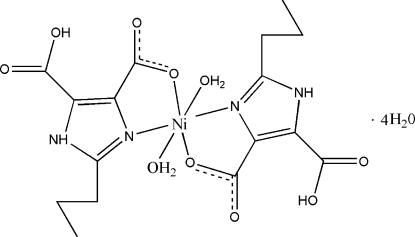


## Experimental

### 

#### Crystal data

[Ni(C_8_H_9_N_2_O_4_)_2_(H_2_O)_2_]·4H_2_O
*M* *_r_* = 561.15
Triclinic, 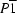

*a* = 10.466 (1) Å
*b* = 10.5829 (11) Å
*c* = 11.3011 (13) Å
α = 81.585 (1)°
β = 83.580 (1)°
γ = 86.869 (2)°
*V* = 1229.5 (2) Å^3^
*Z* = 2
Mo *K*α radiation
μ = 0.86 mm^−1^
*T* = 298 K
0.48 × 0.40 × 0.33 mm

#### Data collection

Bruker SMART 1000 CCD area-detector diffractometer
Absorption correction: multi-scan (*SADABS*; Bruker, 2007 ▶) *T*_min_ = 0.682, *T*_max_ = 0.764
6402 measured reflections
4280 independent reflections
2986 reflections with *I* > 2σ(*I*)
*R*_int_ = 0.026

#### Refinement

*R*[*F* ^2^ > 2σ(*F* ^2^)] = 0.066
*wR*(*F* ^2^) = 0.208
*S* = 1.05
4280 reflections
358 parameters
H-atom parameters constrained
Δρ_max_ = 0.75 e Å^−3^
Δρ_min_ = −1.16 e Å^−3^

### 

Data collection: *SMART* (Bruker, 2007 ▶); cell refinement: *SAINT* (Bruker, 2007 ▶); data reduction: *SAINT*; program(s) used to solve structure: *SHELXS97* (Sheldrick, 2008 ▶); program(s) used to refine structure: *SHELXL97* (Sheldrick, 2008 ▶); molecular graphics: *PLATON* (Spek, 2009 ▶); software used to prepare material for publication: *SHELXTL* (Sheldrick, 2008 ▶).

## Supplementary Material

Crystal structure: contains datablocks I, global. DOI: 10.1107/S1600536810025237/lh5048sup1.cif

Structure factors: contains datablocks I. DOI: 10.1107/S1600536810025237/lh5048Isup2.hkl

Additional supplementary materials:  crystallographic information; 3D view; checkCIF report

## Figures and Tables

**Table 1:** Hydrogen-bond geometry (Å, °)

*D*—H⋯*A*	*D*—H	H⋯*A*	*D*⋯*A*	*D*—H⋯*A*
N2—H2⋯O13^i^	0.86	1.91	2.745 (7)	162
N4—H4⋯O12	0.86	1.88	2.734 (7)	171
O3—H3⋯O2	0.82	1.65	2.466 (6)	176
O7—H7⋯O6	0.82	1.70	2.523 (6)	180
O9—H9*C*⋯O4^ii^	0.85	2.11	2.960 (6)	174
O9—H9*D*⋯O8^iii^	0.85	1.96	2.807 (6)	173
O10—H10*C*⋯O4^iv^	0.85	1.87	2.724 (6)	177
O10—H10*D*⋯O11^v^	0.85	1.83	2.675 (7)	177
O11—H11*C*⋯O1^vi^	0.85	2.06	2.904 (6)	172
O11—H11*C*⋯O2^vi^	0.85	2.62	3.197 (7)	127
O11—H11*D*⋯O6^i^	0.85	1.99	2.830 (6)	172
O12—H12*C*⋯O14	0.85	1.84	2.676 (10)	166
O12—H12*D*⋯O3^vi^	0.85	2.07	2.904 (7)	167
O13—H13*C*⋯O11^vii^	0.85	2.23	2.889 (9)	134
O13—H13*D*⋯O8	0.85	2.44	3.068 (9)	131
O14—H14*G*⋯O13	0.85	1.99	2.488 (11)	117
O14—H14*H*⋯O14^viii^	0.85	1.54	2.355 (17)	160
O14—H14*F*⋯O5^ix^	0.85	2.19	2.778 (9)	127

Symmetry codes: (i) 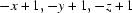; (ii) 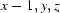; (iii) 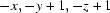; (iv) 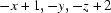; (v) 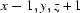; (vi) 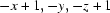; (vii) 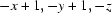; (viii) 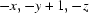; (ix) 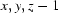.

**Fig. 1.**
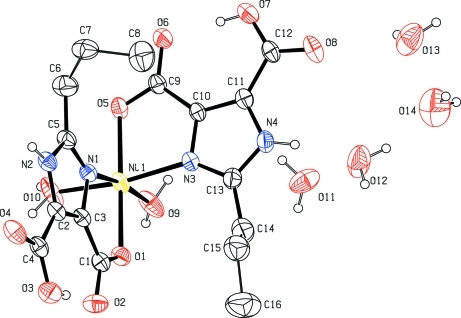
The assymetric unit of the title compound, showing the atomic numbering scheme. Non-H atoms are shown with 30% probability displacement ellipsoids. H atoms bonded to C atoms are not shown. The disorder is not shown.

**Fig. 2.**
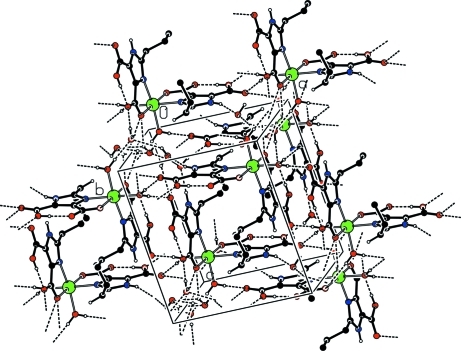
Part of the crystal structure showing O—H···O and N—H···O hydrogen bonds as dashed lines. Neither the disorder in the complex nor the H atoms which are not involved in hydrogen bonds are shown.

## supplementary crystallographic information

### Comment

Recently, imidazole-4,5-dicarboxylic acid (H_3_IDC) with two nitrogen and four oxygen atoms has drawn great interest in coordination chemistry due to the fact that H_3_IDC can be deprotonated to form H_2_IDC^-^, HIDC^2-^ and IDC^3-^ anions at different pH values. H_3_IDC has been widely used to coordinate with metal salts to obtain a series of MOFs with different structures and useful properties (Zou *et al.* , 2006; Li *et al.* , 2006; Liu *et al.* , 2004; Sun *et al.* , 2005), Therefore, we chose H_3_pimda to obtain a new Ni^II^ complex, whose structure is be reported herein.

As illustrated in Fig. 1, the title compound contains an Ni^II^ ion, coordinated by two mono-deprotonated H_2_pimda^-^ anions and two coordinated water molecules in a slightly distorted octahedral geometry. Four solvent water molecules complete the formula unit. The dihedral angle between the two imidazole rings is 95.11 (17)°. In the crystal structure, a three-dimensional framework is formed by intermolecular O-H···O and N-H···O hydrogen bonds involving the solvent water molecules, coordinated water molecules, carboxy O atoms and the protonated N atoms of H_3_pimda ligands. The propyl groups of each H_3_pimda ligands are disordered over two sets of sites with refined occupancies of 0.50 (2):0.50 (2) and 0.762 (11):0.238 (11). In one water solvent molecule, one of the H atoms was refined as disordered over two sites with equall occupancies.

### Experimental

A mixture of NiNO_3_ (0.5 mmol, 0.06 g) and 2-propyl-1*H*-imidazole-4,5-dicarboxylic acid (0.5 mmol, 0.99 g) in 15 ml of C_3_H_7_NO solution was sealed in an autoclave equipped with a Teflon liner (20 ml) and then heated at 433k for 4 days. Crystals of the title compound were obtained by slow evaporation of the solvent at room temperature.

### Refinement

C and N bound H atoms were placed in calculated positions and were treated as riding on the parent C or N atoms with C—H = 0.96-0.97 Å, N—H = 0.86 Å, and with *U*_iso_(H) = 1.2 *U*_eq_(C, N) or 1.5 *U*_eq_(C_methyl_). H atoms of the water molecules were located in a difference Fourier map and were allowed to ride on the parent atom, with O-H = 0.85 Å and *U*_iso_(H)=1.2 *U*_eq_.

### Crystal data

**Table:**

[Ni(C_8_H_9_N_2_O_4_)_2_(H_2_O)_2_]·4H_2_O	*Z* = 2
*M**_r_* = 561.15	*F*(000) = 588
Triclinic, *P*1	*D*_x_ = 1.516 Mg m^−^^3^
Hall symbol: -P 1	Mo *K*α radiation, λ = 0.71073 Å
*a* = 10.466 (1) Å	Cell parameters from 2051 reflections
*b* = 10.5829 (11) Å	θ = 2.5–23.9°
*c* = 11.3011 (13) Å	µ = 0.86 mm^−^^1^
α = 81.585 (1)°	*T* = 298 K
β = 83.580 (1)°	Block, green
γ = 86.869 (2)°	0.48 × 0.40 × 0.33 mm
*V* = 1229.5 (2) Å^3^	

### Data collection

**Table:**

Bruker SMART 1000 CCD area-detector diffractometer	4280 independent reflections
Radiation source: fine-focus sealed tube	2986 reflections with *I* > 2σ(*I*)
graphite	*R*_int_ = 0.026
φ and ω scans	θ_max_ = 25.0°, θ_min_ = 1.8°
Absorption correction: multi-scan (*SADABS*; Bruker, 2007)	*h* = −12→12
*T*_min_ = 0.682, *T*_max_ = 0.764	*k* = −11→12
6402 measured reflections	*l* = −10→13

### Refinement

**Table:**

Refinement on *F*^2^	Primary atom site location: structure-invariant direct methods
Least-squares matrix: full	Secondary atom site location: difference Fourier map
*R*[*F*^2^ > 2σ(*F*^2^)] = 0.066	Hydrogen site location: inferred from neighbouring sites
*wR*(*F*^2^) = 0.208	H-atom parameters constrained
*S* = 1.05	*w* = 1/[σ^2^(*F*_o_^2^) + (0.0945*P*)^2^ + 3.581*P*] where *P* = (*F*_o_^2^ + 2*F*_c_^2^)/3
4280 reflections	(Δ/σ)_max_ = 0.001
358 parameters	Δρ_max_ = 0.75 e Å^−^^3^
0 restraints	Δρ_min_ = −1.16 e Å^−^^3^

### Special details

**Table:**

Geometry. All e.s.d.'s (except the e.s.d. in the dihedral angle between two l.s. planes) are estimated using the full covariance matrix. The cell e.s.d.'s are taken into account individually in the estimation of e.s.d.'s in distances, angles and torsion angles; correlations between e.s.d.'s in cell parameters are only used when they are defined by crystal symmetry. An approximate (isotropic) treatment of cell e.s.d.'s is used for estimating e.s.d.'s involving l.s. planes.
Refinement. Refinement of *F*^2^ against ALL reflections. The weighted *R*-factor wR and goodness of fit *S* are based on *F*^2^, conventional *R*-factors *R* are based on *F*, with *F* set to zero for negative *F*^2^. The threshold expression of *F*^2^ > σ(*F*^2^) is used only for calculating *R*-factors(gt) etc. and is not relevant to the choice of reflections for refinement. *R*-factors based on *F*^2^ are statistically about twice as large as those based on *F*, and *R*- factors based on ALL data will be even larger.

### Fractional atomic coordinates and isotropic or equivalent isotropic displacement parameters (Å^2^)

**Table:**

	*x*	*y*	*z*	*U*_iso_*/*U*_eq_	Occ. (<1)
Ni1	0.15302 (7)	0.21144 (7)	0.80787 (6)	0.0439 (3)	
N1	0.3486 (4)	0.2176 (4)	0.8285 (4)	0.0403 (10)	
N2	0.5487 (4)	0.2256 (4)	0.8689 (4)	0.0449 (11)	
H2	0.6187	0.2557	0.8847	0.054*	
N3	0.1723 (4)	0.2837 (4)	0.6268 (4)	0.0432 (11)	
N4	0.1924 (5)	0.3537 (5)	0.4338 (4)	0.0520 (13)	
H4	0.2055	0.3536	0.3573	0.062*	
O1	0.2132 (4)	0.0190 (4)	0.7957 (4)	0.0502 (10)	
O2	0.3797 (4)	−0.1188 (4)	0.8133 (4)	0.0621 (12)	
O3	0.6045 (4)	−0.1139 (4)	0.8582 (4)	0.0596 (11)	
H3	0.5301	−0.1123	0.8414	0.089*	
O4	0.7386 (4)	0.0255 (4)	0.8992 (4)	0.0598 (12)	
O5	0.1072 (4)	0.4049 (4)	0.8174 (3)	0.0493 (10)	
O6	0.0993 (5)	0.5962 (4)	0.7061 (4)	0.0590 (11)	
O7	0.1250 (5)	0.6826 (4)	0.4849 (4)	0.0625 (12)	
H7	0.1167	0.6548	0.5568	0.094*	
O8	0.1656 (4)	0.6062 (5)	0.3144 (4)	0.0671 (13)	
O9	−0.0354 (4)	0.1738 (5)	0.7901 (5)	0.0746 (15)	
H9C	−0.0961	0.1264	0.8235	0.090*	
H9D	−0.0686	0.2430	0.7563	0.090*	
O10	0.1162 (5)	0.1683 (5)	0.9898 (4)	0.0798 (17)	
H10C	0.1598	0.1079	1.0266	0.096*	
H10D	0.0495	0.1848	1.0361	0.096*	
O11	0.9033 (5)	0.2266 (5)	0.1288 (5)	0.0849 (17)	
H11C	0.8673	0.1570	0.1577	0.102*	
H11D	0.8986	0.2744	0.1835	0.102*	
O12	0.2218 (7)	0.3264 (7)	0.1953 (5)	0.110 (2)	
H12C	0.1862	0.3693	0.1375	0.132*	
H12D	0.2694	0.2670	0.1686	0.132*	
O13	0.2630 (6)	0.6526 (7)	0.0459 (6)	0.112 (2)	
H13C	0.2576	0.7035	−0.0191	0.135*	
H13D	0.1980	0.6641	0.0960	0.135*	
O14	0.1006 (8)	0.4916 (9)	0.0385 (7)	0.152 (3)	
H14G	0.1650	0.5203	−0.0085	0.182*	
H14F	0.0605	0.4436	0.0024	0.182*	0.50
H14H	0.0326	0.5153	0.0054	0.182*	0.50
C1	0.3287 (5)	−0.0078 (5)	0.8114 (5)	0.0436 (13)	
C2	0.4061 (5)	0.0981 (5)	0.8310 (5)	0.0394 (12)	
C3	0.5317 (5)	0.1013 (5)	0.8560 (5)	0.0417 (13)	
C4	0.6338 (6)	−0.0005 (6)	0.8721 (5)	0.0466 (14)	
C5	0.4375 (5)	0.2931 (5)	0.8528 (5)	0.0457 (14)	
C6	0.4222 (7)	0.4317 (7)	0.8555 (8)	0.072 (2)	
H6A	0.4286	0.4444	0.9379	0.086*	0.50 (2)
H6B	0.3345	0.4560	0.8394	0.086*	0.50 (2)
H6'A	0.4762	0.4594	0.9105	0.086*	0.50 (2)
H6'B	0.3334	0.4576	0.8773	0.086*	0.50 (2)
C7	0.5053 (15)	0.5269 (15)	0.7781 (18)	0.069 (6)	0.50 (2)
H7A	0.5949	0.5062	0.7896	0.082*	0.50 (2)
H7B	0.4838	0.6115	0.7996	0.082*	0.50 (2)
C8	0.484 (2)	0.525 (3)	0.645 (2)	0.093 (8)	0.50 (2)
H8A	0.4726	0.4389	0.6324	0.139*	0.50 (2)
H8B	0.5580	0.5582	0.5940	0.139*	0.50 (2)
H8C	0.4092	0.5771	0.6261	0.139*	0.50 (2)
C7'	0.4676 (19)	0.4840 (19)	0.718 (2)	0.068 (6)	0.50 (2)
H7'1	0.5570	0.4575	0.6994	0.082*	0.50 (2)
H7'2	0.4170	0.4455	0.6665	0.082*	0.50 (2)
C8'	0.4540 (19)	0.627 (2)	0.689 (2)	0.100 (8)	0.50 (2)
H8'1	0.3664	0.6540	0.7096	0.150*	0.50 (2)
H8'2	0.4775	0.6522	0.6047	0.150*	0.50 (2)
H8'3	0.5094	0.6655	0.7345	0.150*	0.50 (2)
C9	0.1183 (5)	0.4772 (6)	0.7179 (5)	0.0443 (13)	
C10	0.1527 (5)	0.4136 (5)	0.6109 (5)	0.0380 (12)	
C11	0.1660 (5)	0.4595 (6)	0.4900 (5)	0.0431 (13)	
C12	0.1541 (5)	0.5891 (6)	0.4234 (5)	0.0470 (14)	
C13	0.1946 (6)	0.2498 (6)	0.5181 (6)	0.0553 (16)	
C14	0.2084 (10)	0.1182 (8)	0.4929 (7)	0.087 (3)	
H14A	0.1637	0.1128	0.4232	0.104*	0.762 (11)
H14B	0.1647	0.0643	0.5605	0.104*	0.762 (11)
H14C	0.2409	0.0704	0.5641	0.104*	0.238 (11)
H14D	0.2791	0.1187	0.4296	0.104*	0.238 (11)
C15	0.3406 (12)	0.0638 (11)	0.4702 (11)	0.088 (4)	0.762 (11)
H15A	0.3896	0.0756	0.5354	0.106*	0.762 (11)
H15B	0.3823	0.1091	0.3964	0.106*	0.762 (11)
C16	0.3405 (16)	−0.0808 (12)	0.4595 (14)	0.128 (6)	0.762 (11)
H16A	0.2931	−0.1248	0.5296	0.192*	0.762 (11)
H16B	0.4275	−0.1150	0.4530	0.192*	0.762 (11)
H16C	0.3010	−0.0919	0.3892	0.192*	0.762 (11)
C15'	0.121 (4)	0.030 (4)	0.449 (3)	0.086 (11)	0.238 (11)
H15C	0.0543	0.0036	0.5125	0.103*	0.238 (11)
H15D	0.1707	−0.0455	0.4292	0.103*	0.238 (11)
C16'	0.058 (4)	0.093 (4)	0.338 (4)	0.110 (15)	0.238 (11)
H16D	0.0276	0.1778	0.3492	0.165*	0.238 (11)
H16E	−0.0124	0.0433	0.3268	0.165*	0.238 (11)
H16F	0.1205	0.0965	0.2684	0.165*	0.238 (11)

### Atomic displacement parameters (Å^2^)

**Table:**

	*U*^11^	*U*^22^	*U*^33^	*U*^12^	*U*^13^	*U*^23^
Ni1	0.0360 (4)	0.0492 (5)	0.0421 (4)	0.0037 (3)	−0.0052 (3)	0.0065 (3)
N1	0.038 (2)	0.040 (2)	0.041 (2)	0.004 (2)	−0.0074 (19)	−0.0003 (19)
N2	0.036 (2)	0.047 (3)	0.050 (3)	−0.003 (2)	−0.011 (2)	0.004 (2)
N3	0.045 (3)	0.045 (3)	0.039 (3)	0.000 (2)	−0.007 (2)	0.000 (2)
N4	0.059 (3)	0.060 (3)	0.036 (3)	0.008 (3)	−0.008 (2)	−0.006 (2)
O1	0.049 (2)	0.046 (2)	0.054 (2)	−0.0064 (19)	−0.0120 (19)	0.0011 (18)
O2	0.061 (3)	0.042 (2)	0.084 (3)	0.004 (2)	−0.009 (2)	−0.012 (2)
O3	0.050 (3)	0.055 (3)	0.072 (3)	0.016 (2)	−0.007 (2)	−0.009 (2)
O4	0.039 (2)	0.067 (3)	0.067 (3)	0.010 (2)	−0.007 (2)	0.011 (2)
O5	0.052 (2)	0.058 (3)	0.035 (2)	0.0139 (19)	−0.0061 (17)	−0.0037 (18)
O6	0.078 (3)	0.050 (3)	0.049 (2)	0.011 (2)	−0.012 (2)	−0.0061 (19)
O7	0.075 (3)	0.050 (3)	0.059 (3)	0.003 (2)	−0.012 (2)	0.008 (2)
O8	0.060 (3)	0.084 (3)	0.047 (3)	0.009 (2)	−0.003 (2)	0.016 (2)
O9	0.041 (2)	0.081 (3)	0.089 (4)	−0.010 (2)	−0.015 (2)	0.039 (3)
O10	0.066 (3)	0.103 (4)	0.050 (3)	0.043 (3)	0.011 (2)	0.028 (3)
O11	0.076 (4)	0.083 (4)	0.098 (4)	−0.030 (3)	0.025 (3)	−0.040 (3)
O12	0.133 (6)	0.133 (6)	0.070 (4)	0.049 (4)	−0.022 (4)	−0.042 (4)
O13	0.094 (5)	0.138 (6)	0.122 (5)	0.009 (4)	−0.050 (4)	−0.049 (4)
O14	0.146 (7)	0.185 (9)	0.124 (7)	0.007 (6)	−0.015 (5)	−0.024 (6)
C1	0.040 (3)	0.046 (3)	0.043 (3)	0.000 (3)	−0.005 (2)	0.000 (2)
C2	0.038 (3)	0.040 (3)	0.037 (3)	0.004 (2)	−0.002 (2)	0.002 (2)
C3	0.039 (3)	0.042 (3)	0.040 (3)	0.003 (2)	−0.001 (2)	0.004 (2)
C4	0.041 (3)	0.053 (4)	0.039 (3)	0.008 (3)	0.002 (2)	0.004 (3)
C5	0.041 (3)	0.044 (3)	0.051 (3)	0.004 (3)	−0.012 (3)	0.000 (3)
C6	0.061 (4)	0.056 (4)	0.102 (6)	0.003 (3)	−0.032 (4)	−0.008 (4)
C7	0.054 (9)	0.052 (9)	0.103 (14)	−0.008 (7)	−0.022 (9)	−0.008 (8)
C8	0.067 (12)	0.12 (2)	0.096 (17)	−0.009 (12)	−0.022 (12)	−0.009 (14)
C7'	0.052 (10)	0.059 (11)	0.094 (15)	−0.008 (8)	−0.010 (11)	−0.008 (11)
C8'	0.091 (14)	0.084 (15)	0.119 (17)	0.010 (11)	−0.010 (12)	0.000 (12)
C9	0.041 (3)	0.048 (3)	0.043 (3)	0.006 (3)	−0.008 (2)	−0.001 (3)
C10	0.033 (3)	0.043 (3)	0.037 (3)	0.002 (2)	−0.007 (2)	0.000 (2)
C11	0.033 (3)	0.052 (3)	0.043 (3)	−0.001 (2)	−0.005 (2)	−0.001 (3)
C12	0.036 (3)	0.057 (4)	0.044 (3)	0.000 (3)	−0.007 (2)	0.007 (3)
C13	0.065 (4)	0.056 (4)	0.045 (3)	0.006 (3)	−0.010 (3)	−0.010 (3)
C14	0.123 (8)	0.074 (5)	0.061 (5)	0.017 (5)	−0.014 (5)	−0.007 (4)
C15	0.101 (9)	0.081 (8)	0.079 (7)	0.003 (7)	−0.002 (6)	−0.009 (6)
C16	0.162 (15)	0.080 (9)	0.133 (13)	0.006 (9)	0.028 (11)	−0.024 (8)
C15'	0.10 (3)	0.08 (2)	0.07 (2)	0.00 (2)	0.004 (19)	−0.006 (18)
C16'	0.12 (4)	0.10 (3)	0.10 (3)	0.01 (3)	0.02 (3)	0.00 (2)

### Geometric parameters (Å, °)

**Table:**

Ni1—O10	2.038 (4)	C5—C6	1.472 (9)
Ni1—N3	2.069 (4)	C6—C7	1.485 (17)
Ni1—O9	2.071 (4)	C6—C7'	1.60 (2)
Ni1—N1	2.092 (4)	C6—H6A	0.9700
Ni1—O5	2.092 (4)	C6—H6B	0.9700
Ni1—O1	2.118 (4)	C6—H6'A	0.9700
N1—C5	1.334 (7)	C6—H6'B	0.9700
N1—C2	1.368 (7)	C7—C8	1.55 (3)
N2—C5	1.348 (7)	C7—H7A	0.9700
N2—C3	1.368 (7)	C7—H7B	0.9700
N2—H2	0.8600	C8—H8A	0.9600
N3—C13	1.322 (8)	C8—H8B	0.9600
N3—C10	1.366 (7)	C8—H8C	0.9600
N4—C13	1.347 (8)	C7'—C8'	1.50 (3)
N4—C11	1.367 (8)	C7'—H7'1	0.9700
N4—H4	0.8600	C7'—H7'2	0.9700
O1—C1	1.253 (7)	C8'—H8'1	0.9600
O2—C1	1.262 (7)	C8'—H8'2	0.9600
O3—C4	1.290 (7)	C8'—H8'3	0.9600
O3—H3	0.8200	C9—C10	1.469 (8)
O4—C4	1.227 (7)	C10—C11	1.376 (7)
O5—C9	1.262 (7)	C11—C12	1.471 (8)
O6—C9	1.255 (7)	C13—C14	1.459 (10)
O7—C12	1.294 (8)	C14—C15	1.479 (14)
O7—H7	0.8200	C14—C15'	1.51 (4)
O8—C12	1.212 (7)	C14—H14A	0.9700
O9—H9C	0.8500	C14—H14B	0.9700
O9—H9D	0.8501	C14—H14C	0.9700
O10—H10C	0.8500	C14—H14D	0.9700
O10—H10D	0.8500	C15—C16	1.552 (16)
O11—H11C	0.8500	C15—H15A	0.9700
O11—H11D	0.8500	C15—H15B	0.9700
O12—H12C	0.8500	C16—H16A	0.9600
O12—H12D	0.8500	C16—H16B	0.9600
O13—H13C	0.8500	C16—H16C	0.9600
O13—H13D	0.8500	C15'—C16'	1.52 (5)
O14—H14G	0.8500	C15'—H15C	0.9700
O14—H14F	0.8500	C15'—H15D	0.9700
O14—H14H	0.8500	C16'—H16D	0.9600
C1—C2	1.474 (8)	C16'—H16E	0.9600
C2—C3	1.378 (8)	C16'—H16F	0.9600
C3—C4	1.482 (8)		
			
O10—Ni1—N3	170.23 (19)	C7—C6—H6'B	117.7
O10—Ni1—O9	89.5 (2)	C7'—C6—H6'B	111.2
N3—Ni1—O9	87.80 (18)	H6'A—C6—H6'B	109.4
O10—Ni1—N1	89.03 (19)	C6—C7—C8	109.0 (17)
N3—Ni1—N1	95.10 (18)	C6—C7—H7A	109.9
O9—Ni1—N1	170.79 (18)	C8—C7—H7A	109.9
O10—Ni1—O5	90.89 (18)	C6—C7—H7B	109.9
N3—Ni1—O5	79.85 (16)	C8—C7—H7B	109.9
O9—Ni1—O5	92.39 (19)	H7A—C7—H7B	108.3
N1—Ni1—O5	96.72 (17)	C8'—C7'—C6	113.1 (18)
O10—Ni1—O1	90.27 (18)	C8'—C7'—H7'1	109.0
N3—Ni1—O1	99.18 (17)	C6—C7'—H7'1	109.0
O9—Ni1—O1	91.48 (18)	C8'—C7'—H7'2	109.0
N1—Ni1—O1	79.43 (16)	C6—C7'—H7'2	109.0
O5—Ni1—O1	175.97 (16)	H7'1—C7'—H7'2	107.8
C5—N1—C2	106.1 (4)	C7'—C8'—H8'1	109.5
C5—N1—Ni1	143.1 (4)	C7'—C8'—H8'2	109.5
C2—N1—Ni1	110.3 (3)	H8'1—C8'—H8'2	109.5
C5—N2—C3	108.5 (5)	C7'—C8'—H8'3	109.5
C5—N2—H2	125.7	H8'1—C8'—H8'3	109.5
C3—N2—H2	125.7	H8'2—C8'—H8'3	109.5
C13—N3—C10	106.5 (5)	O6—C9—O5	124.3 (5)
C13—N3—Ni1	142.9 (4)	O6—C9—C10	119.7 (5)
C10—N3—Ni1	110.6 (3)	O5—C9—C10	116.0 (5)
C13—N4—C11	108.6 (5)	N3—C10—C11	109.6 (5)
C13—N4—H4	125.7	N3—C10—C9	118.3 (5)
C11—N4—H4	125.7	C11—C10—C9	132.1 (5)
C1—O1—Ni1	114.9 (4)	N4—C11—C10	105.0 (5)
C4—O3—H3	109.5	N4—C11—C12	122.5 (5)
C9—O5—Ni1	115.1 (4)	C10—C11—C12	132.4 (6)
C12—O7—H7	109.5	O8—C12—O7	121.5 (6)
Ni1—O9—H9C	141.0	O8—C12—C11	120.6 (6)
Ni1—O9—H9D	106.2	O7—C12—C11	117.8 (5)
H9C—O9—H9D	108.2	N3—C13—N4	110.2 (5)
Ni1—O10—H10C	118.9	N3—C13—C14	124.8 (6)
Ni1—O10—H10D	130.0	N4—C13—C14	124.8 (6)
H10C—O10—H10D	108.5	C13—C14—C15	117.4 (9)
H11C—O11—H11D	108.6	C13—C14—C15'	133.9 (16)
H12C—O12—H12D	108.7	C15—C14—C15'	106.0 (16)
H13C—O13—H13D	110.1	C13—C14—H14A	107.9
H14G—O14—H14F	108.6	C15—C14—H14A	107.9
H14G—O14—H14H	108.5	C13—C14—H14B	107.9
O1—C1—O2	123.8 (5)	C15—C14—H14B	107.9
O1—C1—C2	116.6 (5)	H14A—C14—H14B	107.2
O2—C1—C2	119.6 (5)	C13—C14—H14C	104.5
N1—C2—C3	109.8 (5)	C15'—C14—H14C	106.3
N1—C2—C1	118.5 (5)	C13—C14—H14D	104.7
C3—C2—C1	131.7 (5)	H14C—C14—H14D	105.7
N2—C3—C2	105.3 (5)	C14—C15—C16	111.6 (11)
N2—C3—C4	122.9 (5)	C14—C15—H15A	109.3
C2—C3—C4	131.8 (5)	C16—C15—H15A	109.3
O4—C4—O3	123.8 (5)	C14—C15—H15B	109.3
O4—C4—C3	119.7 (6)	C16—C15—H15B	109.3
O3—C4—C3	116.5 (5)	H15A—C15—H15B	108.0
N1—C5—N2	110.3 (5)	C14—C15'—C16'	113 (3)
N1—C5—C6	126.2 (5)	C14—C15'—H15C	109.0
N2—C5—C6	123.4 (5)	C16'—C15'—H15C	109.0
C5—C6—C7	123.6 (9)	C14—C15'—H15D	109.0
C5—C6—H6A	106.4	C16'—C15'—H15D	109.0
C7—C6—H6A	106.4	H15C—C15'—H15D	107.8
C5—C6—H6B	106.4	C15'—C16'—H16D	109.5
C7—C6—H6B	106.4	C15'—C16'—H16E	109.5
H6A—C6—H6B	106.5	H16D—C16'—H16E	109.5
C5—C6—H6'A	111.6	C15'—C16'—H16F	109.5
C7'—C6—H6'A	112.5	H16D—C16'—H16F	109.5
H6B—C6—H6'A	131.5	H16E—C16'—H16F	109.5
C5—C6—H6'B	111.7		
			
O10—Ni1—N1—C5	−83.4 (6)	C2—N1—C5—C6	177.5 (6)
N3—Ni1—N1—C5	87.7 (6)	Ni1—N1—C5—C6	−12.2 (11)
O5—Ni1—N1—C5	7.4 (6)	C3—N2—C5—N1	−0.6 (6)
O1—Ni1—N1—C5	−173.9 (7)	C3—N2—C5—C6	−177.5 (6)
O10—Ni1—N1—C2	86.6 (4)	N1—C5—C6—C7	−121.2 (12)
N3—Ni1—N1—C2	−102.3 (4)	N2—C5—C6—C7	55.2 (13)
O5—Ni1—N1—C2	177.4 (3)	N1—C5—C6—C7'	−89.1 (10)
O1—Ni1—N1—C2	−3.8 (3)	N2—C5—C6—C7'	87.3 (10)
O9—Ni1—N3—C13	−82.1 (7)	C5—C6—C7—C8	64.8 (16)
N1—Ni1—N3—C13	89.1 (7)	C7'—C6—C7—C8	5.8 (18)
O5—Ni1—N3—C13	−174.9 (7)	C5—C6—C7'—C8'	176.0 (15)
O1—Ni1—N3—C13	9.1 (7)	C7—C6—C7'—C8'	−50.6 (17)
O9—Ni1—N3—C10	95.9 (4)	Ni1—O5—C9—O6	−177.5 (5)
N1—Ni1—N3—C10	−92.8 (4)	Ni1—O5—C9—C10	4.1 (6)
O5—Ni1—N3—C10	3.1 (3)	C13—N3—C10—C11	−1.3 (6)
O1—Ni1—N3—C10	−172.9 (3)	Ni1—N3—C10—C11	180.0 (3)
O10—Ni1—O1—C1	−85.5 (4)	C13—N3—C10—C9	176.6 (5)
N3—Ni1—O1—C1	97.0 (4)	Ni1—N3—C10—C9	−2.1 (6)
O9—Ni1—O1—C1	−175.0 (4)	O6—C9—C10—N3	−179.8 (5)
N1—Ni1—O1—C1	3.5 (4)	O5—C9—C10—N3	−1.3 (7)
O10—Ni1—O5—C9	179.0 (4)	O6—C9—C10—C11	−2.5 (9)
N3—Ni1—O5—C9	−4.1 (4)	O5—C9—C10—C11	176.0 (5)
O9—Ni1—O5—C9	−91.4 (4)	C13—N4—C11—C10	0.0 (7)
N1—Ni1—O5—C9	89.9 (4)	C13—N4—C11—C12	−178.7 (5)
Ni1—O1—C1—O2	176.4 (4)	N3—C10—C11—N4	0.8 (6)
Ni1—O1—C1—C2	−2.3 (6)	C9—C10—C11—N4	−176.7 (6)
C5—N1—C2—C3	−0.6 (6)	N3—C10—C11—C12	179.3 (6)
Ni1—N1—C2—C3	−174.4 (4)	C9—C10—C11—C12	1.8 (10)
C5—N1—C2—C1	177.9 (5)	N4—C11—C12—O8	1.2 (9)
Ni1—N1—C2—C1	4.1 (6)	C10—C11—C12—O8	−177.1 (6)
O1—C1—C2—N1	−1.3 (7)	N4—C11—C12—O7	178.0 (5)
O2—C1—C2—N1	179.9 (5)	C10—C11—C12—O7	−0.4 (9)
O1—C1—C2—C3	176.7 (5)	C10—N3—C13—N4	1.3 (7)
O2—C1—C2—C3	−2.1 (9)	Ni1—N3—C13—N4	179.3 (5)
C5—N2—C3—C2	0.2 (6)	C10—N3—C13—C14	−173.9 (7)
C5—N2—C3—C4	−177.9 (5)	Ni1—N3—C13—C14	4.2 (12)
N1—C2—C3—N2	0.2 (6)	C11—N4—C13—N3	−0.8 (7)
C1—C2—C3—N2	−177.9 (5)	C11—N4—C13—C14	174.3 (7)
N1—C2—C3—C4	178.1 (5)	N3—C13—C14—C15	−98.7 (10)
C1—C2—C3—C4	0.0 (10)	N4—C13—C14—C15	86.8 (10)
N2—C3—C4—O4	0.7 (8)	N3—C13—C14—C15'	103 (2)
C2—C3—C4—O4	−177.0 (6)	N4—C13—C14—C15'	−72 (2)
N2—C3—C4—O3	179.5 (5)	C13—C14—C15—C16	173.4 (9)
C2—C3—C4—O3	1.8 (9)	C15'—C14—C15—C16	−22.4 (18)
C2—N1—C5—N2	0.7 (6)	C13—C14—C15'—C16'	48 (4)
Ni1—N1—C5—N2	171.0 (4)	C15—C14—C15'—C16'	−112 (3)

### Hydrogen-bond geometry (Å, °)

**Table:**

*D*—H···*A*	*D*—H	H···*A*	*D*···*A*	*D*—H···*A*
N2—H2···O13^i^	0.86	1.91	2.745 (7)	162
N4—H4···O12	0.86	1.88	2.734 (7)	171
O3—H3···O2	0.82	1.65	2.466 (6)	176
O7—H7···O6	0.82	1.70	2.523 (6)	180
O9—H9C···O4^ii^	0.85	2.11	2.960 (6)	174
O9—H9D···O8^iii^	0.85	1.96	2.807 (6)	173
O10—H10C···O4^iv^	0.85	1.87	2.724 (6)	177
O10—H10D···O11^v^	0.85	1.83	2.675 (7)	177
O11—H11C···O1^vi^	0.85	2.06	2.904 (6)	172
O11—H11C···O2^vi^	0.85	2.62	3.197 (7)	127
O11—H11D···O6^i^	0.85	1.99	2.830 (6)	172
O12—H12C···O14	0.85	1.84	2.676 (10)	166
O12—H12D···O3^vi^	0.85	2.07	2.904 (7)	167
O13—H13C···O11^vii^	0.85	2.23	2.889 (9)	134
O13—H13D···O8	0.85	2.44	3.068 (9)	131
O14—H14G···O13	0.85	1.99	2.488 (11)	117
O14—H14H···O14^viii^	0.85	1.54	2.355 (17)	160
O14—H14F···O5^ix^	0.85	2.19	2.778 (9)	127

Symmetry codes: (i) −*x*+1, −*y*+1, −*z*+1; (ii) *x*−1, *y*, *z*; (iii) −*x*, −*y*+1, −*z*+1; (iv) −*x*+1, −*y*, −*z*+2; (v) *x*−1, *y*, *z*+1; (vi) −*x*+1, −*y*, −*z*+1; (vii) −*x*+1, −*y*+1, −*z*; (viii) −*x*, −*y*+1, −*z*; (ix) *x*, *y*, *z*−1.
